# ﻿Back to the future: A preserved specimen validates the presence of *Molossuspretiosus* (Molossidae, Chiroptera) in Honduras

**DOI:** 10.3897/zookeys.1196.116144

**Published:** 2024-03-22

**Authors:** Manfredo A. Turcios-Casco, Vinícius Cardoso Cláudio, Thomas E. Lee Jr

**Affiliations:** 1 Asociación para la Sostenibilidad e Investigación Científica en Honduras (ASICH), Barrio La Granja, entre 28 y 29 calle, Comayagüela M.D.C., Francisco Morazán, Tegucigalpa, Honduras Asociación para la Sostenibilidad e Investigación Científica en Honduras (ASICH) Tegucigalpa Honduras; 2 Laboratório de Etnoconservacao e Áreas Protegidas, Universidade Estadual de Santa Cruz, Ilhéus, BA, Brazil Universidade Estadual de Santa Cruz Ilhéus Brazil; 3 Fundação Oswaldo Cruz, Fiocruz Mata Atlântica, 22713-560, Rio de Janeiro, Brazil Fundação Oswaldo Cruz, Fiocruz Mata Atlântica Rio de Janeiro Brazil; 4 Department of Biology, Box 27868, Abilene Christian University, Abilene, Texas, 79699, USA Abilene Christian University Texas United States of America

**Keywords:** Bat diversity, Caribbean islands, Central America, distribution, Islas de la Bahía, morphology

## Abstract

*Molossuspretiosus* is a molossid bat that has been thought to exist in Honduras. While some authors have suggested its range extends all the way to Mexico, others have placed its northernmost distribution in Nicaragua. We present evidence, based on one specimen collected in 2005, confirming the presence of this species in the Caribbean of Honduras within the Islas de la Bahía department. This discovery increases the count of known species within this family to 18 in the country and raises the total bat species count for Honduras to 114. We recommend a detailed study of historical specimens to confirm the identification of species that may have been misidentified as well as a thorough examination of molossids distributed in northern Honduras.

## ﻿Introduction

Currently, 113 bat species has been reported for Honduras (Turcios-Casco et al. 2020b; Mora et al. 2021), new records which include: *Lasiuruscinereus* (Palisot de Beauvois, 1796), *Lasiurusegregius* (Peters, 1870), *Neoeptesicusbrasiliensis* (Desmarest, 1819), *Balantiopteryxio* Thomas, 1904, *Vampyriscusnymphaea* (Thomas, 1909), *Nyctinomopsaurispinosus* (Peale, 1848), *N.macrotis* (Gray, 1840), *Hylonycterisunderwoodi* Thomas, 1903, *Chirodermagorgasi* Handley, 1960, *Diaemusyoungii* (Jentink 1893), *Nataluslanatus* Tejedor, 2005, *Cynomopsmexicanus* (Jones and Genoways 1967) and *Centronycteriscentralis* Thomas, 1912 (Espinal and Mora 2012; Mora 2012; Divoll and Buck 2013; Mora et al. 2014; Espinal et al. 2016, 2021; Mora et al. 2016; Turcios-Casco and Medina-Fitoria 2019; Turcios-Casco et al. 2020a, b). One of the major issues with Honduran bat studies is that there are several historically-preserved specimens that have not been verified yet. For example, museum specimens of *Nataluslanatus* and *Cynomopsmexicanus* reported by Turcios-Casco et al. (2020b) were collected in 1963 and 1967, respectively. Collecting and recording efforts have increased in the past 15 years, raising the number of bat species to 113 (Turcios-Casco et al. 2020b). Turcios-Casco et al. (2020b) and Mora et al. (2021), expect additional species to occur based on their wide distributions in Central America these include: *Cormurabrevirostris* (Wagner, 1843), *Lampronycterisbrachyotis* (Dobson, 1879), *Trinycterisnicefori* (Sanborn, 1949), *Mesophyllamacconnelli* (Thomas, 1901), *Molossuscoibensis* J. A. Allen, 1904, *Molossuspretiosus* Miller, 1902 and *Thyropteradiscifera* (Lichtenstein & Peters, 1855). Historical collections from Honduras in international museums remain poorly assessed, and verification of the identification of many species (e.g., in the genera *Natalus*, *Molossus*, *Eumops* and phyllostomines) not only of bats (e.g., rodents such as *Peromyscus*, shrews such as *Cryptotis*) remains unclarified.

One of the most contentious bat families in Central America is Molossidae, because their overlapping external characters [see diagnoses in Loureiro et al. (2018, 2019, 2020)] make field identification difficult, and also because most of their echolocation calls have not been verified within their distribution (B. Miller, pers. comm.). Therefore, taxonomic identification of Central American molossids remains problematic and unresolved.

Among molossids, the Miller’s Mastiff Bat, *Molossuspretiosus*, has been considered widely distributed (Loureiro et al. 2019), and even though some authors consider it to be distributed far north as Mexico and southward to Brazil (e.g., Simmons 2005; Medina-Fitoria 2014; Cláudio et al. 2018; Díaz et al. 2021), the species was not included in Mexico by Ramírez-Pulido et al. (2014). Additionally, historical specimens in the Global Information Biodiversity Facility (GBIF.org 2023) database that have been identified as *M.pretiosus* were recorded from Belize and Mexico. The majority of the specimens identified as *M.pretiosus* in northern Central America, especially from Mexico and Belize, must be confirmed, as they could have been confused with other species of *Molossus* (B. Miller, pers. comm.). This occurrence was supported by Díaz et al. (2021) who included *M.pretiosus* in the bat checklist for Mexico and Costa Rica but not for Belize. Burgin et al. (2020) also confirm the species in Mexico and Belize. In contrast, other authors considered Nicaragua as the northernmost country within its range (e.g., Loureiro et al. 2019; Jennings et al. 2000; Simmons and Cirranello 2023). As described above, there is controversy over the distribution of *M.pretiosus* in northern Mesoamerica. As part of an effort to further investigate museum specimens from Honduras, we present evidence that confirms the occurrence of *M.pretiosus* on the Caribbean Island of Roatán in northern Honduras.

## ﻿Materials and methods

### ﻿Preserved specimens and description of the locality

On 6 March 2005, one specimen of *M.pretiosus* (ACUNHC 1034) was found on the beach shore of Fantasy Island Resort in Roatán, within the Islas de la Bahía department in northern Honduras. The skull and the skeleton were deposited in the Abilene Christian University Natural History Collection (ACUNHC-Mammal). Roatán is the largest island (40 km long and 8 km wide) within the Islas de la Bahía department; it reaches approximately 300 m a.s.l. in elevation and is mostly covered with tropical dry forests and mangroves along the shore; private properties are very common all along the island (Goode et al. 2020).

### ﻿Morphological description

For the identification of the specimen, we mainly followed Cláudio et al. (2018) to compare Latin American samples, as well as Nogueira et al. (2008) and Loureiro et al. (2018, 2019, 2020) for taking the following measurements: forearm length (FA) , greatest length of skull including incisors (GLS) , condylobasal length (CBL) , condylocanine length (CCL) , postorbital breadth (PB) , zygomatic breadth (ZB) , braincase breadth (BB) , mastoid breadth (MB) , maxillary toothrow length (MTL) , breadth across molars (BM) and breadth across canines (BC). Calipers accurate to the nearest 0.01 mm were used to take the measurements. Additional publications were consulted to help us describe the qualitative and quantitative characteristics of ACUNHC 1034: DeBlase and Martin (1981), Dolan (1989), Gregorin and Taddei (2000), Jennings et al. (2000), Loureiro et al. (2018, 2019, 2020) and Díaz et al. (2021).

### ﻿Distribution

**Figure 1.**
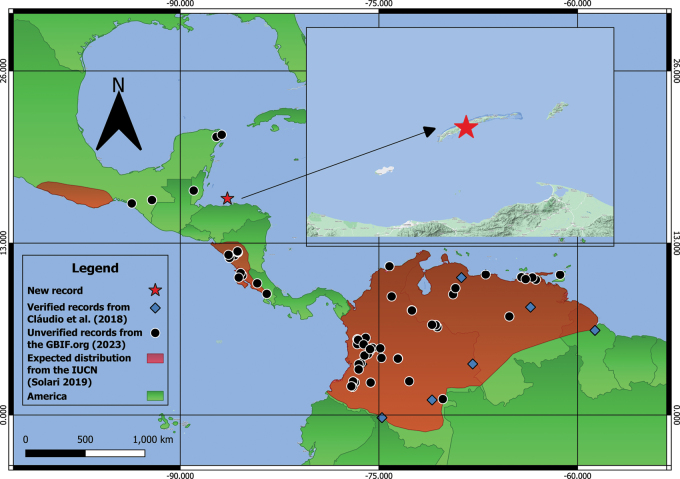
Distribution map of *Molossuspretiosus*. We considered the records mentioned by Cláudio et al. (2018) as verified, and we included in the distribution other records based on the GBIF.org (2023) database. In addition, we overlap these records with the expected distribution of the species based on the IUCN (Solari 2019).

Data from the Global Biodiversity Information Facility (GBIF.org; accessed in September 2023) of all preserved *M.pretiosus* specimens was downloaded and analyzed to define the distribution of the species. These records included both verified specimens (Gregorin and Taddei 2000; Eger 2008; Cláudio et al. 2018; Loureiro et al. 2019, 2020) and specimens recorded in the GBIF that need to be further verified (Fig. 1). Additionally, the range of the species was contrasted with the data presented by the IUCN, International Union for Conservation of Nature (Solari 2019).

## ﻿Results

### ﻿ Molossus pretiosus

Taxon classificationAnimaliaChiropteraMolossidae

Miller, 1902

42A07879-1621-5B9E-B7A3-16DF19EF4AB3

#### Material examined.

Honduras • 1 ♀; Roatán, Fantasy Island Resort, Islas de la Bahía department; 16°21'30"N, 86°26'9"W; 4 m a.s.l.; 6 March 2005; Thomas Lee leg; dead specimen found on the ground; ACUNHC 1034.

#### Description.

**Figure 2.**
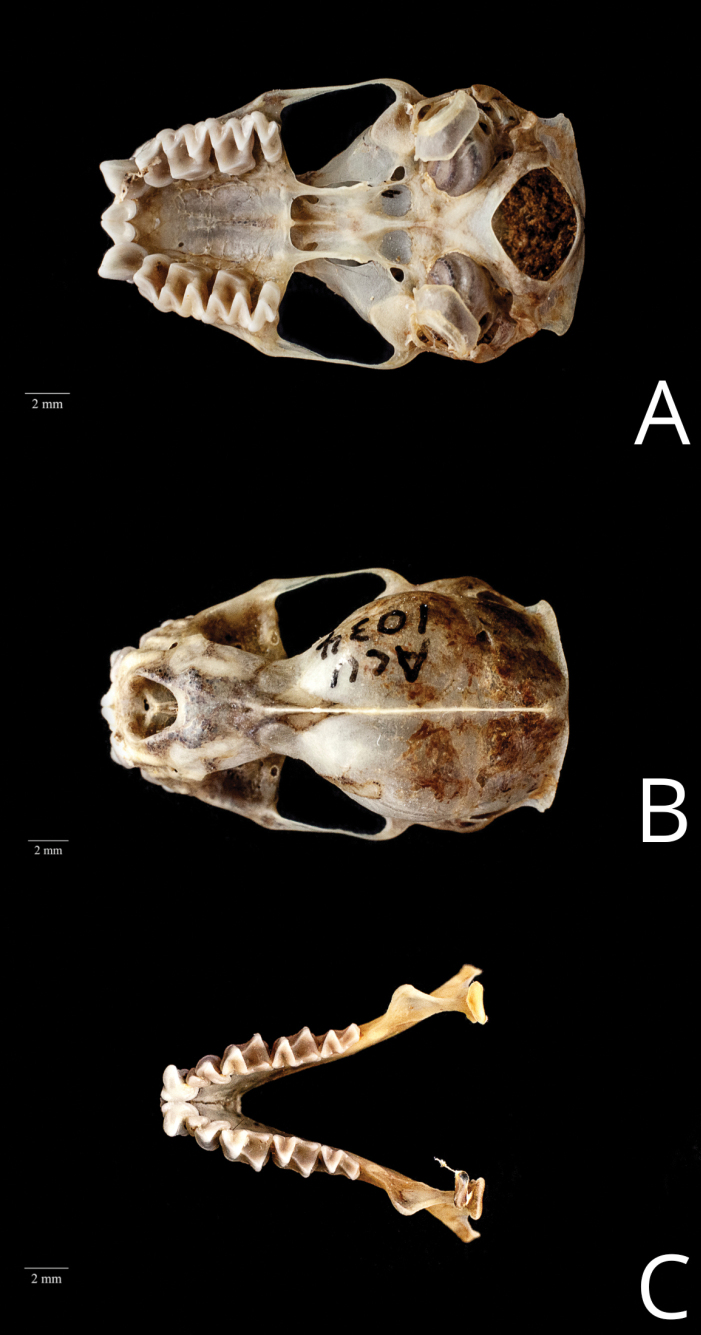
Skull (**A, B**) and mandible (**C**) of Molossuspretiosus (ACUNHC 1034) from Roatán Island. A ventral view (squarish occipital complex) B dorsal view (note the well-developed bulging braincase and sagittal crest C dorsal view of the upper mandible (M3 molars with a V-shaped pattern). Credits of the photos are to Nil Santana (ACUNHC 1034).

Given that the skin (ACUNHC 1034) was desiccated when discovered, no description is available for the fur. As a result, the identification of the specimen primarily relies on characteristics of the skull (Table 1). The rostrum is short and quadrangular in shape. There is no projection over the nasal cavity by the nasal process of the premaxilla; therefore, the nasal process is undeveloped. The skull presented a squarish occipital complex (Fig. 2A) because of the significant size and angling of the lambdoidal crests. The skull exhibits distinct features, including a well-developed bulging braincase and sagittal crest (Fig. 2B), as well as a deep basioccipital pit with a ridge. There is a noticeable crest between the basisphenoid and basioccipital pits. Infraorbital foramen opened laterally, when viewing from the front. Additionally, the M3 molars display cup-like structures with a V-shaped pattern (Fig. 2C). The elongated upper incisors extend beyond the canines and the tips and are not in contact. The forearm measurement in the dried skin of the ACUNHC 1034 specimen is recorded as 45.08 mm. This measurement matches the range observed in Central American specimens (44.3–45.9 mm) (Jennings et al. 2000).

#### Comparisons.

In comparison to the other species of *Molossus* that occur in Honduras, *Molossusnigricans* Miller, 1902 is larger than *M.pretiosus*, the FA of the former varies from 47.2–54.5 mm in females and GLS from 20.1–22.6 mm in females (Loureiro et al. 2019). *Molossusalvarezi* (González-Ruíz, Ramírez-Pulido & Arroyo-Cabrales, 2011) is medium-sized and may overlap in some measurements, but it does not have a well-developed sagittal crest; it has pincer-like upper incisors converging at the tips, and the occipital region is triangular (González-Ruíz et al. 2011; Díaz et al. 2021). *Molossusmolossus* (Pallas, 1766) is usually smaller with FA ranging from 36.4–42.6 mm in females and GLS from 15.6–18.6 mm in females (Loureiro et al. 2018). In addition to the other two species that occur in Honduras, *Molossusbondae* J. A. Allen, 1904 and *Molossusaztecus* Saussure, 1860 have upper incisors as pincer-like with convergent tips, but those of *M.pretiosus* are larger than those of *M.bondae* (FA <43 mm) (Loureiro et al. 2019; Jennings et al. 2000), and *M.aztecus* differs in having its basisphenoid pits with a moderate depth (Díaz et al. 2021), and is currently only known from western Honduras (McCarthy et al. 1993; Turcios-Casco et al. 2021).

## ﻿Discussion

In addition to *M.alvarezi*, *M.aztecus*, *M.bondae*, *M.molossus* and *M.nigricans*, we present the record of a sixth *Molossus* species to Honduras, *M.pretiosus*. This brings the total number of molossids known to occur in Honduras to 18 [*Cynomopsgreenhalli* Goodwin, 1958; *C.mexicanus*; *Eumopsauripendulus* (Shaw, 1800); *Eumopsferox* (Gundlach, 1961); *Eumopshansae* Sanborn, 1932; *Eumopsnanus* (Miller, 1900); *Eumopsunderwoodi* (Goodwin, 1940); *N.aurispinosus*; *Nyctinomopslaticaudatus* (É. Geoffroy, 1805); *N.macrotis*; *Promopscentralis* Thomas, 1915; and *Tadaridabrasiliensis* (I. Geoffroy, 1824)]; and increases the current list of bat species for Honduras to 114 (see Turcios-Casco et al. 2020b). There are still poorly known molossids in the country; *T.brasiliensis*, one of the most common and widespread species of molossid (Kunz et al. 1995), is known from two official records, one in western Honduras in Ocotepeque and another in the central region of the country in Francisco Morazán (McCarthy et al. 1993; Turcios-Casco et al. 2021). In addition, *M.aztecus* is only known from historical records from La Paz in western Honduras, supported by the revision of McCarthy et al. (1993) of museum specimens misidentified as *M.bondae* by Goodwin (1942).

Molossidae is still an understudied mammalian group in Honduras. One of the major issues of studying the group in the country is that many of them lack a robust and verified database of their echolocation calls, besides the limited sampling that has been done on bat acoustics since 1999 in Honduras (B. Miller pers. comm.). Reasons for this lack of information also include the small number of researchers interested in the family in Honduras, acoustic research being a recent addition to bat monitoring, and the relatively new use of canopy nets (B. Miller. pers. comm.). The natural history and ecological behaviour of molossids, which typically forage above mist nets, also present challenges to their study (Simmons 2005; Cláudio et al. 2018; Díaz et al. 2021). The under-utilization and lack of revision of historical mammalian specimens from Honduras present significant obstacles to accurately describing the biogeography of these animals. Misidentifications resulting from outdated taxonomy can impede efforts to elucidate the historical ranges of species, hindering our understanding of past ecosystems and potentially influencing modern conservation strategies.

The record of *M.pretiosus* presented herein for Honduras fills the gap in the northern portion of its distribution (Fig. 1) and indicates that it may be present in other regions of northern Mesoamerica. Since there is still some uncertainty about the northern limits of the distribution of the species, we strongly recommend revisiting museum specimens of *M.pretiosus* in northern Mesoamerica (e.g., Mexico and Belize) and verifying calls of molossids in Central America to identify other characteristics (e.g., bioacoustics) for molossid species identification as well as analysing molecular data whenever possible. Additionally, special attention must be given to the population in the Caribbean islands of Honduras because molossids in this region could probably be misidentified due to the overlap of external characters. To avoid the misidentification of specimens, we recommend the use of a larger set of morphological traits during field identification, such as forearm length, shape and length of upper indictors, fur colour and pattern of banding (see Loureiro et al. 2018, 2019, 2020; Díaz et al. 2021). Also, the collection and deposition of voucher specimens in scientific collections are encouraged in order to verify and confirm field identifications. Finally, the proper preparation of museum specimens such as skull, skeletons, skins, and tissues for further molecular studies is fundamental, especially for problematic groups like Molossidae.

**Table 1.** Comparison of cranial measurements (see Material and methods for abbreviations) and forearm length (FA) of *Molossuspretiosus* specimens along its distribution in Latin America. Specimens and locations as follow: 1. Cláudio et al. (2018), 2. Nogueira et al. (2008), 3. Gregorin and Taddei (2000) and 4. Dolan (1989).

Measurements	ACUNHC 1034 (this study)	Bahía, Brazil^1^	Minas Gerais, Brazil^2^	Mato Grosso do Sul, Brazil^3^	Costa Rica^4^	Nicaragua^4^
**FA**	45.0	46.3	43.6–47.2	42.6–45.5	43.4–46.0	41.6–45.9
**GLS**	18.3	19.8	19.1–20.4	19.2–19.6	19.7–20.9	18.8–20.8
**CBL**	17.4	18.2	17.5–18.5	–	17.5–18.1	16.4–18.6
**CCL**	17.1	18.1	–	–	–	–
**PB**	3.8	4.0	4.1–4.6	–	–	–
**BB**	9.0	10.0	9.8–10.5	9.7–9.9	9.6–10.6	9.7–10.6
**ZB**	10.9	12.3	–	–	–	–
**MB**	10.4	10.8	11.9–13.5	–	–	–
**MTL**	7.2	7.0	7.0–7.5	7.2–7.3	6.8–7.1	6.3–7.4
**BM**	8.1	8.8	8.5–9.7	8.8–9.2	8.3–9.0	8.5–9.3
**BC**	4.5	4.8	5.0–5.6		5.0–5.3	4.8–5.5

## Supplementary Material

XML Treatment for Molossus pretiosus

## ﻿Additional information

### Author ORCIDs

Manfredo A. Turcios-Casco https://orcid.org/0000-0002-3198-3834

Vinícius Cardoso Cláudio https://orcid.org/0000-0002-3438-911X

Thomas E. Lee Jr https://orcid.org/0000-0001-5185-5568

### Data availability

All information pertaining to the validation of the findings in this study can be found in the main text.
